# Integrative analysis of miRNA and gene expression reveals regulatory networks in tamoxifen-resistant breast cancer

**DOI:** 10.18632/oncotarget.11136

**Published:** 2016-08-09

**Authors:** Tejal Joshi, Daniel Elias, Jan Stenvang, Carla L. Alves, Fei Teng, Maria B. Lyng, Anne E. Lykkesfeldt, Nils Brünner, Jun Wang, Ramneek Gupta, Christopher T. Workman, Henrik J. Ditzel

**Affiliations:** ^1^ Sino-Danish Breast Cancer Research Centre, University of Copenhagen, Copenhagen, Denmark; ^2^ Center for Biological Sequence Analysis, Department of Systems Biology, Technical University of Denmark, Kongens Lyngby, Denmark; ^3^ Department of Cancer and Inflammation Research, Institute of Molecular Medicine, University of Southern Denmark, Odense, Denmark; ^4^ Section of Molecular Disease Biology, Department of Veterinary Disease Biology, University of Copenhagen, Copenhagen, Denmark; ^5^ BGI (Beijing Genomics Institute), Beishan Industrial Zone, Shenzhen, China; ^6^ Breast Cancer Group, Cell Death and Metabolism, Danish Cancer Society Research Center, Copenhagen, Denmark; ^7^ Department of Oncology, Odense University Hospital, Odense, Denmark

**Keywords:** miRNAs, breast cancer, miRNA-mediated gene regulation, endocrine resistance, antihormonal therapy

## Abstract

Tamoxifen is an effective anti-estrogen treatment for patients with estrogen receptor-positive (ER+) breast cancer, however, tamoxifen resistance is frequently observed. To elucidate the underlying molecular mechanisms of tamoxifen resistance, we performed a systematic analysis of miRNA-mediated gene regulation in three clinically-relevant tamoxifen-resistant breast cancer cell lines (TamRs) compared to their parental tamoxifen-sensitive cell line. Alterations in the expression of 131 miRNAs in tamoxifen-resistant vs. parental cell lines were identified, 22 of which were common to all TamRs using both sequencing and LNA-based quantitative PCR technologies. Although the target genes affected by the altered miRNA in the three TamRs differed, good agreement in terms of affected molecular pathways was observed. Moreover, we found evidence of miRNA-mediated regulation of *ESR1, PGR1, FOXM1* and *14-3-3* family genes. Integrating the inferred miRNA-target relationships, we investigated the functional importance of 2 central genes, SNAI2 and FYN, which showed increased expression in TamR cells, while their corresponding regulatory miRNA were downregulated. Using specific chemical inhibitors and siRNA-mediated gene knockdown, we showed that both SNAI2 and FYN significantly affect the growth of TamR cell lines. Finally, we show that a combination of 2 miRNAs (miR-190b and miR-516a-5p) exhibiting altered expression in TamR cell lines were predictive of treatment outcome in a cohort of ER+ breast cancer patients receiving adjuvant tamoxifen mono-therapy. Our results provide new insight into the molecular mechanisms of tamoxifen resistance and may form the basis for future medical intervention for the large number of women with tamoxifen-resistant ER+ breast cancer.

## INTRODUCTION

Tamoxifen is a widely used therapy for estrogen receptor alpha-positive (ER+) breast cancer, and has been shown to be highly effective in the adjuvant setting, with 28% reduction in mortality at 15 years of follow-up [1]. In addition to the adjuvant setting, anti-estrogen treatment with tamoxifen is also effective in the metastatic setting and, although not curative, extends survival. Tamoxifen is the recommended treatment modality for premenopausal breast cancer patients and for postmenopausal patients with contraindications for treatment with aromatase inhibitors (AIs). In addition, the side-effects of anti-estrogenic drugs differ and some patients may not be eligible for a given drug due to co-morbidities [2]. It is, therefore, rational to maintain tamoxifen as an adjuvant treatment option for postmenopausal ER+ breast cancer patients, although AIs have been shown to be superior in this group of patients [2]. Although tamoxifen greatly benefits many patients, recurrence occurs in almost all patients with advanced disease and approximately 30% of ER+ patients at 15-years of follow-up despite adequate treatment [1]. Metabolites of tamoxifen, such as 4-hydroxytamoxifen, bind to the estrogen receptor (ER), competing with estrogens and thereby inhibiting transcription of estrogen-responsive genes [3]. Previous studies have shown a loss or decreased expression of ER in ER+ breast cancer patients treated with tamoxifen [4, 5]. Altered ER expression might be linked to genetic and epigenetic changes, such as hypermethylation of CpG islands in 5^’^ regulatory regions of ER, or there may be other genetic events that affect ER expression [6]. For example, mutations localized in the ligand-binding domain of the ER gene (*ESR1*) have been identified in metastatic lesions of patients treated with tamoxifen [7, 8]. Various studies have shown the impact of altered expression of ER-beta, co-activators and co-repressors of ER, IGF1, IGF1R, IGFBP3 and various other growth factors and receptors on tamoxifen resistance [2, 5, 9, 10]. Aberrant activation of alternative pathways of cellular proliferation may also contribute to tamoxifen-resistant tumor growth. Among the major players regulating molecular pathways are short, 21 nucleotide long, non-coding genes called miRNAs that regulate gene expression at the post-transcriptional level. In mammals, more than 60% of protein-coding genes undergo post-transcriptional regulation in the form of degradation or translation inhibition through miRNAs [11, 12]. Due to their potential to regulate mRNAs through tumor-suppressing or -inducing capacities, miRNAs have recently been investigated to determine their contributions to cancer development and progression [12, 13]. Gene regulation by miRNAs often leads to activation or dysregulation of various pathways responsible for the development of drug resistance. For example, the miR-221/222 cluster miRNAs regulate levels of ER and could thereby play a critical role in tamoxifen resistance and in ER+ cancers in general [14, 15]. Furthermore, a recent study has shown that miR-22 negatively regulates ER expression [16]. Apart from ER, restoration of TIMP3 expression via suppression of its regulatory miRNA (miR-222, miR-181b) has been shown to restore tamoxifen sensitivity in a mouse tamoxifen-resistant xenograft model [17]. However, a majority of previous studies have focused on a limited number of miRNAs and have lacked a system-wide view of miRNA-mediated tamoxifen resistance.

In the present study, we investigated miRNA-mediated gene regulation associated with tamoxifen resistance by analyzing a clinically-relevant, isogenic, human breast cancer MCF-7-based tamoxifen-resistant cell line model using small-RNAseq, global LNA-based miRNA quantitative RT-PCR (qPCR) and microarray profiling. Since it is known that miRNA target predictions often suffer a high number of false-positives, we performed integrative inverse-correlation analysis of miRNAs and mRNA expression profiles to distinguish likely functional miRNA-target relationships from spurious computational target predictions. The results provide interesting insights into affected biological processes in tamoxifen resistance. By integrating various lines of evidence related to miRNA-gene regulation, we posit a novel functional miRNA-gene relationship, and a subsequent clinical validation confirmed that some of the miRNA alterations observed in our TamR cell line model correlated with disease outcome in clinical primary tumor samples of breast cancer patients receiving adjuvant tamoxifen mono-therapy.

## RESULTS

### Tamoxifen resistance-associated miRNA alterations in the tamoxifen-resistant cell line model

**Figure 1:**
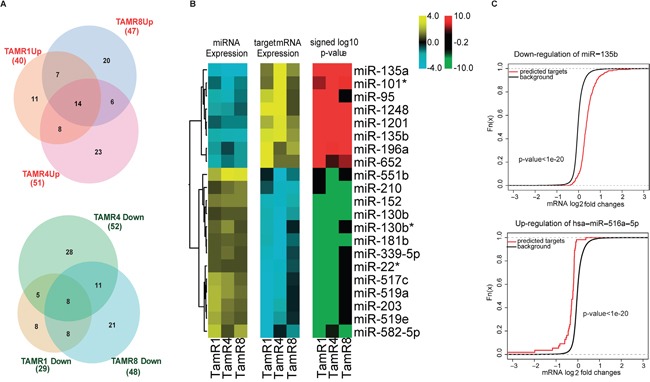
Inverse correlation analysis identified coherent direction of expression changes of miRNAs and their predicted functional targets **A.** Overlaps of differentially-expressed miRNAs in the three TamR vs. MCF-7/S0.5 cell lines, as measured by qPCR assays, are depicted in Venn Euler's diagrams. Circle size corresponds to the number of altered miRNAs in a given cell line. **B.** Heatmaps depict changes of expression common in all the TamR cells relative to MCF-7/S0.5. Log-fold changes of the miRNAs exhibiting altered expression in all resistant cell lines compared to MCF-7/S0.5 (14 upregulated and 8 downregulated) were plotted (panel 1 of the heatmap). Targets of these miRNAs identified using the inverse correlation analysis exhibited an opposite trend in the direction of the altered expression (panel 2), thereby displaying the effect of possible regulation by their miRNAs. **C.** The predicted functional targets of upregulated miRNAs were more downregulated than the non-targets (signed log10 p-values of the Wilcoxon rank-sum test.

miRNA expression profiles of three tamoxifen-resistant breast cancer cell lines (TamR1, TamR4 and TamR8) and the parental tamoxifen-sensitive cell line (MCF-7/S0.5) were analyzed by LNA-based qPCR in biological triplicates. Additionally, miRNA expression profiles for the 4 cell lines were obtained using Illumina's small-RNAseq. Comparison of miRNA expression profiles of the 3 TamRs vs. MCF-7/S0.5 obtained by qPCR identified a common tamoxifen resistance-associated signature consisting of 14 upregulated and 8 downregulated miRNAs (Table 1 and Figure 1A). Moreover, each of the resistant cell lines exhibited altered expression of a large number of miRNAs compared to MCF-7/S0.5 (Supplementary Tables 1 and 2). In total, 131 miRNAs exhibited alterations in expression between the parental and tamoxifen-resistant cell lines.

**Table 1:** Log-fold changes of miRNAs with consistent significantly altered expression across all TamR cell lines relative to MCF-7/S0.5 cell lines (adjusted P < 0.05) using LNA-based qPCR assay

miRNA	TAMR1	TAMR4	TAMR8
miR-101*	−1.02	−3.05	−1.37
miR-1201	−0.94	−1.36	−1.27
miR-1248	−1.68	−1.61	−1.01
miR-652	−1.99	−1.17	−1.64
miR-95	−1.82	−2.45	−1.19
miR-135b	−1.89	−2.16	−2.33
miR-196a	−1.54	−0.71	−1.65
miR-135a	−2.84	−6.16	−4.74
miR-130b	0.75	0.94	0.89
miR-130b*	1.06	1.55	0.88
miR-152	1.29	1.4	1.49
miR-181b	0.89	1.14	0.95
miR-203	2.2	1.34	1.12
miR-210	0.7	1.18	1.49
miR-22*	0.97	0.88	0.82
miR-339-5p	1	1.26	0.8
miR-516a-5p	3.47	2.87	2.46
miR-517c	2.06	1.66	1.06
miR-519a	2.23	1.69	1.2
miR-519e	1.98	1.15	0.97
miR-551b	2.09	5.79	3.51
miR-582-5p	2.43	0.86	1.93

TamR1 and TamR4 shared 13 miRNAs expressed at significantly lower levels than MCF-7/S0.5, including an oncomiR miR-95. TamR1 and TamR8 shared 16, and TamR4 and TamR8 shared 19, miRNAs at lower expression levels than MCF-7/S0.5. In terms of miRNAs exhibiting higher expression in TamRs vs. MCF-7/S0.5 cells, TamR1 and TamR4 shared 22 miRNAs; TamR1 and TamR8 shared 21 miRNAs, including the tumor suppressors miR-181b, oncomiR miR-210 and a known ER regulator miR-18a [18]; and finally, TamR4 and TamR8 shared 20 upregulated miRNAs, including oncomiRs miR-210, miR-203, miR-18a* (Figure 1A and Supplementary Table 1).

**Figure 2:**
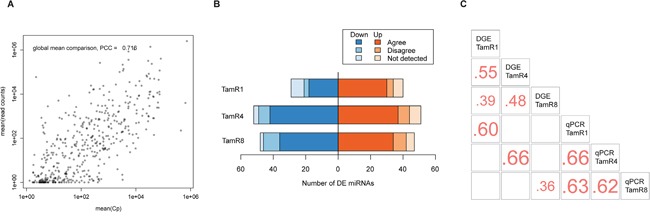
Expression of miRNAs in tamoxifen-resistant and -sensitive cell lines **A.** Global mean expression of miRNAs measured using qPCR and sequencing technologies showed a high overall correlation (r = 0.72). **B.** Agreement between significantly differentially-expressed (DE) miRNAs identified by qPCR vs. RNAseq. The number of up- and downregulated miRNAs discovered by both technologies are shown. **C.** Pearson's correlation coefficients of miRNA fold-changes as measured by qPCR and sequencing platforms. The comparison is based on the set of miRNAs with significant differential expression using qPCR.

Expression levels measured by qPCR and small-RNAseq showed a good overall correlation (Pearson's correlation coefficient, 0.72) between mean Cp-value across cell line replicates and read counts for all cell line types (Figure 2A). A majority of differentially-expressed miRNAs found by qPCR were also detectable using sequencing (Supplementary Table 3), and showed agreement in terms of the direction of expression change. Only a minority of differentially expressed miRNAs using qPCR were not detectable by small-RNAseq. Twenty-eight of the 69 miRNAs exhibiting altered expression in TamR1 by qPCR showed altered expression profiles (absolute log 2-fold change of at least 0.7 in TamR1) by small-RNAseq. Similarly, 58 of the 103 altered miRNAs in TamR4, and 34 of 95 altered miRNAs in TamR8, also exhibited altered expression as determined by small-RNAseq (Figure 2B and Supplementary Tables 3 and 4). Pairwise comparisons of miRNA log2-fold changes for each TamR cell line measured by both technologies showed poor (*r* = 0.36 for TamR8) to good (*r* = 0.66 for TamR4) correlations (Figure 2C).

### Global analysis of miRNA-target relationships

**Figure 3:**
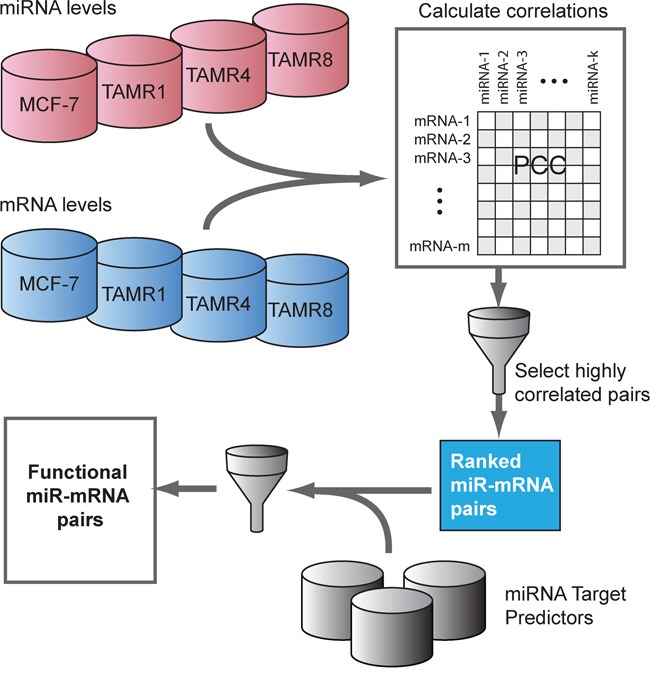
Inverse-correlation analysis of miRNA and mRNA expression data to identify predicted functional miRNA-targets A pairwise correlation matrix for mRNA and miRNA expression levels (Cp values) was constructed from the mean expression across cell line replicates. The top ranking miRNA-mRNA pairs by Pearson correlation coefficient (“PCC”, r ≤ −0.8) were selected and assessed for a computationally-predicted miRNA-target association inferred by more than one miRNA target predictors. Predicted functional targets are the computationally-predicted miRNA-target pairs with high degree of inverse association at expression levels.

To analyze miRNA-mediated regulation in tamoxifen resistance, the expression profiles of 197 miRNAs that exhibited ≥0.7 absolute log2-fold change by qPCR were integrated with global mRNA expression profiles of these same cell lines (Figure 3). Using inverse-correlation analysis on miRNA-mRNA expression profiles, we derived miRNA-target pairs that, in addition to being predicted targets, showed inverse-correlation of miRNA-mRNA levels. Predicted miRNA targets with significant inverse correlations (r ≤ −0.8) of expression with corresponding regulating miRNAs were obtained for each significantly-altered miRNA (Figure 1A). Consistent inverse patterns of differential expression were observed for miRNAs and their predicted functional targets, supporting our hypothesis of miRNA regulation (Figure 1B, panel I, II and III of the heatmap and Supplementary Table 5). For example, predicted functional targets (Supplementary Table 6) of miR-135b showed a stronger tendency toward upregulation compared to the non-targets (p-value < 0.05 for Wilcoxon rank sum test) (Figure 1C).

The significance of miRNA regulation on differentially-expressed mRNAs was measured using odds ratios (Supplementary Table 7), which indicated that 63% of these mRNAs could be accounted for by changes in the expression of one or more regulating miRNAs.

We next determined the fraction of miRNAs included in the qPCR-based miRNA-mRNA inverse-correlation analysis that were also identified by small-RNAseq. Of the total 197 miRNAs included in the inverse-correlation analysis, 118 showed significant p-values (adjusted p-value <0.05) measured by qPCR, and 89 of these miRNAs (75%) showed agreement in the direction of fold-change by sequencing, whereas 11 miRNAs did not agree by small-RNAseq. Eighteen miRNAs (9%) from the above set were not detected by small-RNAseq. Thirty-two of the 79 miRNAs from the 197 miRNA-mRNA inverse-correlation list that did not show significant fold-changes by qPCR exhibited significant fold-change (≥0.7 log2 fold change) by small-RNAseq, thus adding to the overall number of significant miRNA in the miRNA-mRNA inverse-correlation list.

### Functional enrichment of miRNA-regulated genes

miRNA-regulated mRNAs exhibiting altered expression in each of the individual TamR cell lines were investigated for their potential to elucidate molecular mechanisms through miRNA-mediated post-transcriptional regulation. miRNAs exhibiting altered expression in TamR1 were found to regulate mRNAs from cancer-related signaling pathways (FDR < 0.05), such as TGF-beta (FDR < 0.05), MAPK, Wnt, EphrinB-EPHB and ECM-receptor signaling pathways. In TamR4, miRNAs exhibiting altered expression seemed to regulate mRNAs associated with signaling by Aurora kinases (FDR < 0.05), FOXM1 transcription factor network (FDR < 0.05), and cell cycle M Phase and G2/M transition (FDR < 0.05), glypican pathway, FOXA transcription factor network, PDGFR-beta signaling, Kit receptor, axon guidance and PDGF signaling pathway. Finally, miRNAs exhibiting altered expression in TamR8 were found to regulate mRNAs involved in the IFN-gamma pathway, Jak-STAT signaling, plasma membrane ER signaling and neurotrophin signaling pathways, although the analysis of TamR8 did not achieve statistical significance.

We also examined whether these minimally overlapping sets of differentially-expressed functional targets shared any common mechanisms of drug resistance resulting from effects on any specific pathways. miRNA-regulated mRNAs in TamR1 and TamR4 cell lines showed a strong enrichment (FDR< 0.05) of the glypican and IGF1 pathways, PDGFR-beta signaling, IFN-gamma pathway, plasma membrane ER signaling, TGF-beta signaling, nuclear and cytoplasmic SMAD2/3, BMP signaling and class I PI3K signaling events. Although not significant, association to functional categories for miRNA-regulated genes in TamR8 included IFN-gamma pathway, Jak-STAT signaling, plasma membrane ER signaling and neurotrophin signaling pathways. Thus, despite the lack of agreement among resistant cell lines in terms of differentially expressed mRNAs, we observed a good agreement in terms of affected pathways, implying that similar mechanisms of tamoxifen resistance may exist in the distinct resistant cell lines.

### miRNA-mediated regulation of *ESR1* and *PGR* expression

**Figure 4:**
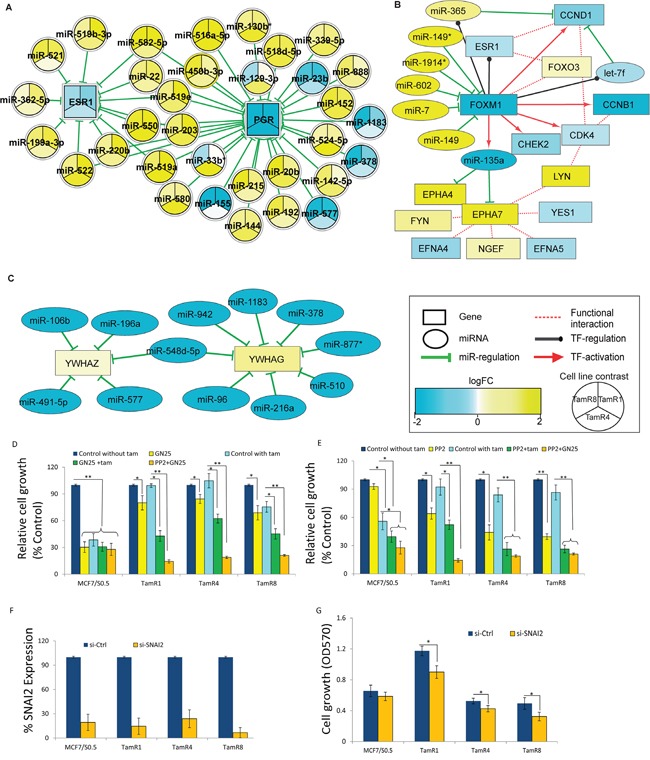
miRNA-mediated gene regulation events in tamoxifen-resistant cell lines and functional evaluation of their importance in tamoxifen resistance **A.** Consistent downregulation of estrogen receptor-alpha and progesterone receptor by several miRNAs exhibiting altered expression in TamR1, TamR4 or TamR8 relative to MCF-7/S0.5, as indicated using node colors. **B.** FOXM1 expression is regulated by a number of miRNAs in TamR4. Suppressed expression of FOXM1 positively correlated with the expression of its transcriptional targets, including miR-135a, let-7f and miR-365. **C.** YWHAG and YWHAZ appeared to be under miRNA regulation in TamR8. Color and intensity of the nodes correspond to the extent of fold-changes observed in the relevant comparison. **D.** Growth of MCF-7/S0.5 and TamR cell lines following treatment with the selective P53-SNAIL binding inhibitor GN25 (3μM) or its solvent, DMSO (Control), or **E.** the selective SKF inhibitor PP2 (2.5 μM) or its solvent, ethanol (Control) in medium with or without tamoxifen (1μM). Cells were also treated with combined GN25 and PP2. Cell growth was determined at 72 hrs using a colorimetric assay. **F.** Expression of SNAI2 in MCF7 or TamR cell lines following SNAI2-specific or control siRNA transfection as measured by qRT-PCR. **G.** Growth of cells in tamoxifen-containing medium measured 4 days after siRNA transfection, * *p*<0.01, ** p<0.001.

We further examined the involvement of miRNAs on two of the key genes in breast cancers, *ESR1* and *PGR*. Putative modulators of *ESR1* and *PGR* expression were identified by inverse correlation analysis of miRNA and mRNA profiles (Figure 4A and Supplementary Table 5). Such a combinatorial regulation of *ESR1* and *PGR* levels is strongly evident in TamR1, where their miRNA regulators were expressed at significantly higher levels in the tamoxifen-resistant vs. -sensitive cell line; TamR4 and TamR8 displayed similar patterns, albeit to a lesser extent (Figure 4A). Although both up- and downregulated miRNA-regulators of *ESR1* and *PGR* were identified, higher numbers of the former were observed among the regulating miRNAs, suggesting a combinatorial downregulation of target mRNAs by upregulated miRNAs in TamR cells.

### Gene regulation upstream and downstream of FOXM1 in TamR4

One of the ER-regulator proteins, Forkhead box protein, FOXM1 is known to play an important role in ER+ breast cancer by interacting with *ESR1*. Our inverse correlation analysis identified a number of significantly upregulated miRNA regulators of this gene, consistent with significant downregulation of FOXM1 in TamR4 (Figure 4B). Expression of the known *FOXM1*-regulated mRNAs, including *CHEK2, CCNB1, CDK4* and *CCND1*, as well as miR-135a and let-7f, correlated with *FOXM1* expression in TamR4, but not in TamR1 or TamR8. Moreover, miR-135a was one of the consistently downregulated miRNAs in all TamRs, with lowest levels of miR-135a observed in TamR4. To test whether the reduced miR-135a expression in TamR4 cells was the direct result of low levels of *FOXM1* in these cells compared to the tamoxifen-sensitive MCF-7/S0.5 cells, we performed siRNA knockdown of *FOXM1* and evaluated miR-135a expression. Initially, using quantitative real-time RT-PCR analysis, we confirmed that *FOXM1* showed 3-fold higher expression in MCF-7/S0.5 vs. TamR4 cells, while miR-135a showed >9 fold higher expression in MCF-7/S0.5 vs. TamR4 cells, confirming the findings from the microarray and small RNA-seq analysis (Supplementary Figure 1, top panel). Although siRNA transfection resulted in over 80% knockdown of FoxM1 transcript in both cell lines (Supplementary Figure 1, top panel), no significant alteration of miR-135a expression was observed, suggesting that reduction of the *FOXM1* level in TamR4 alone was not sufficient to alter the miR-135a level (Supplementary Figure 1, lower panel).

### Loss of miRNA regulation for YWHAG and YWHAZ

Our inverse correlation analysis also identified the two 14-3-3 family members YWHAZ and YWHAG upregulated in TamR8 vs. MCF-7/S0.5, but not in TamR1 and TamR4. These genes appeared to be under direct regulation of a relatively small number of miRNAs (Figure 4C), including miR-96, miR-942, miR-378, miR-196a, miR-106b, miR-577, miR-491-5p, which showed significant downregulation and inverse correlation with the YWHAG and YWHAZ expression in TamR8 (adjusted p-value ≤ 0.05).

### Blocking of central miRNA/mRNA tamoxifen resistance-related pathways in TamR cells using specific chemical inhibitors

Among the miRNAs downregulated in TamR vs. MCF7/S0.5 cells were miR-593, miR-342-3p/5p, and miR-33b. Among their predicted targets were SNAI2 (mir-593) and FYN (miR-342-3p/5p and miR-33b), respectively, two genes that showed increased expression in TamR vs. MCF7/S0.5 cells (Supplementary Figure 2). To investigate whether the reduced expression of miR-593/increased expression of SNAI2 had functional significance for tamoxifen resistance, we evaluated the growth of TamR and MCF7/S0.5 cells in the presence or absence of tamoxifen when the SNAIL-P53 binding inhibitor GN25 was added. The results showed that GN25 alone has a moderate effect on TamR cells but, when combined with tamoxifen, dramatically reduces growth, suggesting that inhibition of SNAI2 activity renders TamR cells highly susceptible to tamoxifen (Figure 4D).

Similarly, we evaluated whether the reduced expression of miR-342-3p/5p and miR-33b/increased expression of FYN had functional significance for tamoxifen resistance by evaluating the growth of TamR and MCF7/S0.5 cells in the presence or absence of tamoxifen when the cells were treated with SRC family kinase inhibitor PP2. The results showed that, treatment of TamR cells with PP2 resulted in significant growth inhibition of TamR cells, but no marked effect on the parental cell line (Figure 4E). Interestingly, similar to our observation with SNAI2 inhibitor, PP2 markedly enhanced tamoxifen-induced growth inhibition in cells otherwise resistant to tamoxifen. Moreover, to evaluate whether the 2 genes promote tamoxifen resistance independently or in combination, we treated TamR cells with the combination of PP2 and GN25 and found that the combination resulted in much more potent inhibition than the 2 compounds used separately (Figure 4D and 4E), suggesting that the 2 genes may promote resistance through separate pathways. In the tamoxifen-sensitive parental cell line, treatment with GN25, tamoxifen or combination of the 2 led to comparable and dramatic growth inhibition (Figure 4D), while PP2 treatment did not affect the growth of parental cell lines (Figure 4E).

### siRNA-mediated knockdown of SNAI2 increases tamoxifen-induced growth inhibition in tamoxifen-resistant breast cancer cell lines

To further evaluate whether the reduced expression of mir-593 and corresponding increased expression of its target gene SNAI2 is associated with tamoxifen resistance, we performed gene knockdown of, SNAI2. The result showed that siRNA transfection of breast cancer cell lines led to over 80% reduction in SNAI2 expression as measured by qRT-PCR (Figure 4F). The reduction in SNAI2 resulted in significant reduction of TamR cell growth, while no significant reduction in growth of parental MCF7/S0.5 cells was observed (Figure 4G), suggesting that mir-593 may affect tamoxifen resistance by controlling SNA12 expression.

### miRNA alterations in the cell line model are also seen in breast cancers of patients receiving adjuvant tamoxifen monotherapy

**Figure 5:**
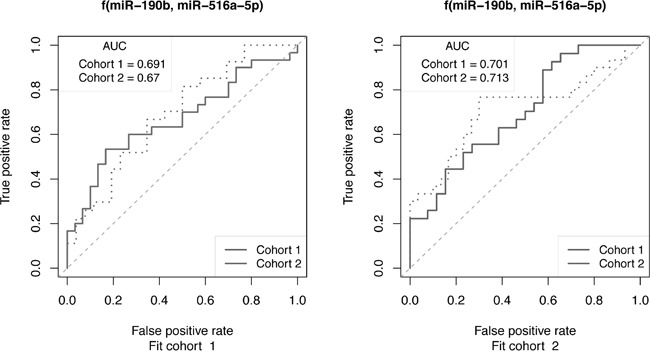
ROC curve analysis to assess the ability of miRNAs to predict outcome of cohorts of ER+ breast cancer patients receiving tamoxifen monotherapy Selected miRNAs that exhibited altered expression in TamRs vs. MCF-7/S0.5 cell lines were also predictive of recurrence following tamoxifen treatment. The 2-miR classifier, miR-190b and miR-516a-5p, was fit on cohorts 1 and 2 (Fit cohort) and then applied to both cohorts independently. Training performance is observed when Fit cohort and test cohorts are the same (dotted lines), while independent test set performance is observed when they differ (solid lines).

We assessed the clinical relevance of our results from the cell line model by comparing the miRNA data therefrom with those of primary tumors from ER+ breast cancer patients receiving adjuvant tamoxifen monotherapy. In this recent study, global miRNA expression in primary tumors of patients who experienced disease recurrence despite tamoxifen treatment were compared those who did not (data set GSE37405) [19]. As outlined in Materials and Methods, the miRNA data from the clinical study consisted of three independent cohorts. Patient classification was designed to separate training performance and independent test performance for all of the single miRNAs and for combinations of miRNAs by fitting a classifier on each cohort independently and testing that classifier on all three cohorts. Prediction performance was assessed by ROC analysis and summarized by the area under the ROC curve (AUC). The analysis showed that as single miRNA classifiers, miR-190b, miR-29b miR-516a-5p and miR-203, were the most promising (Supplementary Figure 3). Improved prognostic power of treatment outcome was observed for the combination of 2 miRNAs (miR-190b and miR-516a-5p) in 2 of 3 patient cohorts (Figure 5). Other combinations, including the 2 miRNAs combinations: miR-190b and miR-29b, miR-190b and miR-516a-5p, and the 3 miRNAs combinations: miR-190b, miR-29b and miR-203; miR-29b, miR-516a-5p and miR-203; miR-190b, miR-516a-5p and miR-29b, miR-190b, miR-516a-5p and miR-203 also exhibited promising prognostic power of treatment outcome (Supplementary Figure 3).

## DISCUSSION

Drug resistance is a major clinical issue in cancer treatment. In this study, we identified molecular mechanisms of tamoxifen resistance in ER+ breast cancer affected by miRNAs and their target mRNAs in a cell line model. To strengthen the regulatory associations of miRNAs and target mRNAs, we utilized inverse expression correlations between miRNAs and their computationally-identified mRNA targets and integrated curated evidence of TF-DNA and protein-protein interaction information to identify relevant miRNA-mediated mRNA regulations. Several miRNA-target relationships that extend beyond the known signaling events associated with tamoxifen resistance in breast cancer were identified.

Genomic heterogeneity is an important factor that complicates the interpretation of results obtained by global analysis of patient tumor material using advanced genomic technologies. This includes evaluation of tamoxifen resistance, which may be the result of different mechanisms in different patient tumors as well as within different areas of a single tumor [20]. Single cell sequencing may overcome this problem, but this technique is not commonly available and thus intratumor heterogeneity and thereby heterogeneity in the resistance mechanisms significantly complicate data interpretation [20]. Previous studies have shown that the TamR cell line model analyzed in the present study displays protein alterations also observed in clinical samples of patients resistant to tamoxifen treatment [21, 22], supporting the clinical relevance of the model. The three individual tamoxifen-resistant cell lines studied may represent individual resistant cell clones within a tumor. In accordance with this, we observed that the distinct TamR cell lines exhibited both shared and unique miRNA and mRNA expression changes. However, despite the uniqueness of miRNAs and mRNAs affected in the individual resistant clones, there was a good overall agreement in the affected molecular pathways.

Adding clinical relevance to our findings, comparison of the miRNA alterations observed in the cell line model to those observed in a large cohort of 152 primary ER+ breast cancer patients treated with tamoxifen [19] showed that in particular the combination of 2 miRNAs (miR-190b and miR-516a-5p) were predictive of treatment outcome in 2 of 3 patient cohorts.

We found that more than 60% of the significantly differentially-expressed genes showed a coherent inverse correlation of expression to their putative miRNA regulators (Supplementary Table 7). In addition, combinatorial regulation by miRNA, where more than one miRNA targets the 3′ UTR of mRNAs, may lead to a stable attenuation of expression of target mRNAs [23]. As an example of combinatorial regulation by miRNA is 14-3-3γ (YWHAG), which frequent overexpression is predictive of poor patient outcome in tamoxifen-resistant breast cancer and functions by activation of the MAPK and PIK pathways [24, 25]. We observed that YWHAG and YWHAZ were upregulated in TamR8, but not in TamR1 or TamR4. Their putative miRNA regulators were significantly downregulated in TamR8 (*p* ≤ 0.05). Bergamaschi *et al*. [24] recently showed that tamoxifen plays a suppressive role on expression of miR-451 in TamR cell lines by inducing loss-of-regulation of YWHAZ due to downregulation of miR-451, its known regulatory miRNA. However, we found no significant over-expression of miR-451 in our study.

Furthermore, regulatory circuits consisting of transcription factors and miRNAs regulate gene expression at both the transcriptional and post-transcriptional levels. Transcription factor *FOXM1*, previously shown to bind the promoter of miR-135a and to positively regulate miR-135a levels [26], showed a downregulation, in agreement with reduced level of miR-135a and increased expression of EPHA4/EPHA7 specifically in TamR4 (Figure 4B). Given the important roles of FOXM1, EPHA4 and EPHA7 on breast cancer outcome [27-29], FOXM1 and miR-135a were good candidates for a siRNA knockdown study. However, we found that reduction of *FOXM1* alone was not sufficient to modulate miR-135a expression in TamR4, and downregulation of miR-135a was dependent on other factors that were not modulated in this siRNA study.

In our experiment, miR-181b was consistently upregulated in all tamoxifen-resistant cell lines. In agreement with this, increased miR-181b expression was also associated with tamoxifen resistance by others [17]. Functional targets of miR-181b appeared to be relevant for breast cancer and drug resistance and were significantly downregulated in our study, including *HEY1, CA2, PIK3R1, LYN, ESR1, JUN, STAT1, MYB, BCL2, CYCS, BAMBI, CTGF* and *SOX9*. MiR-342 was one of the consistently downregulated miRNAs in all resistant cell lines and has been linked with tamoxifen resistance by Cittelly *et al*. [30] We identified 14 predicted functional targets of miR-342-3p/5p, including *FYN, TGFBR1, COL4A6, CDKN1A*, and Ephrins *EPHA4/7* (Supplementary Figure 2). miR-221 and miR-222 are known to be associated with tamoxifen-resistance [14], and our data provided some support for this hypothesis. miR-221 level was slightly increased in TamR1 and TamR4 (log2FC 0.53 and 0.64, respectively, *p* ≤ 0.05), but not in TamR8, whereas miR-222 was downregulated specifically in TamR4 (log2FC −0.51, *p* ≤ 0.05), perhaps as a consequence of a regulatory event upstream of miR-221/222 regulation.

The miRNA-mRNA inverse-correlation analysis identified miR-593, which was downregulated in TamR cell lines vs. MCF7/S0.5, while its predicted target SNAI2 was upregulated. Similarly, miR-342-3p/5p and miR-33b were downregulated in TamR vs. MCF7/S0.5 cells, while their predicted target FYN was upregulated. To assess whether the two miRNA-gene axis were central to the tamoxifen resistance phenotype, we used chemical inhibitors of the two genes and evaluated their influence on TamR growth. Interestingly, GN25, a chemical agent interfering with SNAI2 binding to P53, reduced the growth of all three TamR cell lines only in the presence of tamoxifen, while no effect was observed on TamR growth in the absence of tamoxifen, and its effect on the parental cell line was independent of tamoxifen. This suggests that the reduced mir-593/enhanced SNAI2 expression observed in TamR cells may influence the effect of tamoxifen, and that specific inhibition of SNAI2 function may render the cells susceptible to the growth-inhibiting effects of tamoxifen. Some GN25-mediated growth inhibition of the parental cell line was also observed. While SNAI2 was upregulated in TamR vs. parental cell lines, some expression in the parental cell line was observed. As GN25 treatment may lead to the complete abolition of SNAIL activity, and since SNAI2 is an anti-apoptotic molecule [31], complete abolition of its activity may affect survival of the cells, including the parental cell lines. This suggests that while residual expression of SNAI2 is essential for cell survival, the increased expression observed in tamoxifen-resistant cells may contribute to the resistance phenotype.

Similarly, we performed growth assays of TamR cell lines in the presence of a selective inhibitor of SRC family kinases PP2 and showed that the inhibitor reduced the growth of TamR cells, but had no significant effect on the tamoxifen-sensitive parental cell line, suggesting that the altered miR-342-3p/5p and miR-33b/FYN axis observed in TamR cells is important in the resistance mechanism. TamR cell growth was inhibited both in the presence and absence of tamoxifen, suggesting that FYN acts independently of ER. In line with this, we have recently shown that siRNA knockdown of *FYN* in TamR cells enhanced their susceptibility to the effects of tamoxifen [32]. Although we have not proven that the PP2-mediated growth inhibition of TamR cells was due to inhibition of FYN kinase activity, as the drug targets multiple SRC family kinases at high concentrations, it seems highly plausible since FYN and LYN were the only SRC family kinases that exhibited increased expression, and knockdown of LYN in TamR cells did not affect cell growth [32]. Similarly for GN25, only SNAI2 was upregulated in TamR, and not the other snail proteins, SNAI1 or SNAI3, recognized by GN25. Moreover, using the PP2 and GN25 inhibitors in combination led to a dramatic reduction in cell viability beyond that of either of the two compounds alone, suggesting that the two genes independently contribute to resistance. It may be argued that chemical inhibitors are non-specific and the effect may not be attributed to a specific gene, however, knockdown of *SNAI2* using siRNA transfection resulted in marked reduction in cell growth in TamR cell lines similar to the chemical inhibitors, confirming the importance of the genes in endocrine resistance. In agreement with this, a recent report showed that reduced expression of SNAI2 reduces the aggressiveness of cancer cells [33].

A recent study by Ward *et al*. [34] identified miR-375 as a marker of tamoxifen resistance in a different tamoxifen-resistant cell line model, showing that miR-375 expression level was directly proportional to tamoxifen sensitivity. However, in our study, we observed no miR-375 downregulation and indeed found a significant upregulation in TamR8 (logFold change 1.05, p ≤0.05), perhaps due to differences in the cell models used. Further, of the ten miRNAs with significant downregulation in the tamoxifen-resistance models of Ward *et al*. [34], three were also downregulated in our study (miR-135a, miR-135b, miR-190b), while five were missing or undetectable by our qPCR assays. Among twelve significantly upregulated miRNAs in the same study [34], five were also upregulated in our study in one or more cell lines (miR-551b, miR-519a, miR-521, miR-205 and miR-455-3p), while four were missing or undetectable by our qPCR assays.

Our systematic approach of inferring miRNA-target relationships elucidated important and detailed gene regulation events that may contribute to the development of tamoxifen resistance. Several miRNA-target relationships were identified, many showing significant association with various signaling events previously associated with the development of tamoxifen resistance. Clearly further functional studies are needed to experimentally confirm the identified miRNA-target relationships. While we realize that the observed gene regulation is a combination of miRNA targeting as well as post-transcriptional modifications of the upstream promoters of miRNAs and/or their target genes, our study nonetheless supports the importance of miRNA-mediated gene regulatory events in tamoxifen resistance. Targeted experiments based on our findings will bring insight into tamoxifen resistance in ER+ breast cancer and provide the basis for future medical intervention of ER+ tamoxifen-resistant breast cancer.

## MATERIALS AND METHODS

### Cell lines and standard culture conditions

The human breast cancer cell line MCF-7 was originally received from The Breast Cancer Task Force Cell Culture Bank, Mason Research Institute (Worcester, MA). The MCF-7 cells were gradually adapted to grow in low serum concentration and the tamoxifen-sensitive subline MCF-7/S0.5 [35] was used to establish TamR cell lines by extended treatment with high dose tamoxifen (1 μM), as previously described [21, 36]. The three TamR cell lines, MCF-7/TamR-1 (TamR1), MCF-7/TamR-4 (TamR4), and MCF-7/TamR-8 (TamR8) were derived from distinct colonies grown in culture of MCF-7/S0.5 cells incubated with tamoxifen [22]. The cells were grown in a standard phenol-red-free DMEM:F12 (1:1) medium (21041-025, Gibco, Naerum, Denmark) supplied with 1% heat-inactivated FBS (10270-106, Gibco), 6 ng/ml insulin (I6634, Sigma, St Louis, MO, USA) and 2.5 mM glutamax (35050, Gibco). The standard medium for the TamR cell lines was supplied with 1 μM tamoxifen (T5648, Sigma). MCF-7/S0.5 and the TamR cell lines were passaged once per week and seeded in 1×10^5^ and 1.4×10^5^/T25 flasks, respectively. The cell lines were cultured at 37°C and 5% CO_2_ and kept at low passage numbers throughout the experiments (<10 passages). For authentication of the cell lines DNA fingerprinting by short tandem repeat (STR) analysis (Cell IDTM system, Promega, Roskilde, Denmark) was performed.

### miRNA profiling using quantitative real-time PCR

Total RNA from cell line cultures was extracted and purified using TRIzol (Invitrogen, Naerum, Denmark) and EtOH-precipitated. miRNA qPCR profiling was conducted using miRCURY LNA™ Universal RT microRNA PCR system (Exiqon, Vedbaek, Denmark), per manufacturer's instructions. In brief, 50 ng total RNA was reverse-transcribed in 40 μl reactions on a Bio-RAD S1000 Thermal Cycler (60 min at 42°C, followed by heat-inactivation of the reverse transcriptase for 5 min at 95°C). From the resulting cDNA, 32.5 μl was used for a SYBR green master mix and run in 10 μl real-time amplification on microRNA Ready-to-use PCR, Human panel I+II, V2.R plates (Exiqon, product number 203608) on a Roche LightCycler^®^ 480 real-time PCR system. The applied PCR settings were: 10 min at 95°C, 40 amplification cycles (95°C/10 sec, 60°C/1 min, ramp rate 1.6°C/sec). For each cell line, three biological replicates were analyzed.

### Small RNA library and sequencing

Total RNA (48 μl) from cell line cultures was extracted and purified using a TRIzol reagent kit (Invitrogen) according to the operational manual protocol. Small RNA libraries were generated from the purified RNA using Illumina's Small RNA v1.5 Sample Preparation kit (Illumina, Shanghai, China) according to the manufacturer's guidelines. Briefly, the RNA sample was size fractionated, and 18-30 nt RNA was isolated and purified (6-8 μl). After 5′ and 3′ adaptor ligation, RNA was reverse-transcribed and amplified using 14 PCR cycles of 98°C for 10 sec and 72°C for 15 sec to generate small RNA libraries. The libraries (1 μl) were loaded on an Agilent Technologies 2100 Bioanalyzer to check size and purity, and qPCR to check concentration. Libraries were sequenced on an Illumina Genome Analyzer II and processed with the Illumina pipeline v1.4.0.

### Accession numbers

NCBI Gene Expression Omnibus database accession number for the raw data of miRNA qPCR and miRNA sequencing of MCF-7/S0.5 and the three TAM^R^ cell lines is GSE56411.

### Gene expression arrays

Total RNA was purified from each cell line using TRIzol (Invitrogen) and EtOH-precipitated. Two to 3 independent cultures were used for RNA purifications for each of the TamR cell lines grown separately, and each of the biological replicates (3, 3 and 2, respectively) were individually analyzed on Affymetrix Gene Chip^®^ Human Genome U133 plus 2 arrays (High Wycombe, UK). For MCF-7/S0.5 cells, RNA from 6 independent cultures were purified and arrayed separately. One cycle target labeling and hybridization were performed following manufacturer's instructions and as described by Elias *et al*. [32].

### Analysis of miRNA qPCR data

Primers leading to more than one peak in melting characteristics were identified and annotated via melting curve analysis performed in R. We applied a signal-dependent, non-linear, normalization method similar to that described by Workman *et al*. [37]. The offset amounts were calculated by fitting cubic-spline to the entire dataset and were added to the raw data to obtain desired target distributions. Our normalization approach offered 20% reduction in replicate standard error of means. Subsequently, we used *limma* [38] from Bioconductor package to identify miRNAs with altered expression between tamoxifen-resistant and -sensitive cell lines.

### Analysis of sequencing data

Low-quality 3′ ends of reads were trimmed, and those shorter than 15 nt were eliminated. Prior to alignment, a table was compiled of unique reads with the number of copies of each in a given experiment. These reads were aligned to the subset of mature human miRNA sequences obtained from miRBase [39] September 2010 release, that were covered by the qPCR panels. BLAT search of reads to the reference was applied with no mismatches or gaps allowed in the alignment. Multiple-mapped reads were discarded from further analysis. Normalized read counts per miRNA were analyzed for differential expression using DESeq [40] in Bioconductor R environment.

### Analysis of microarray data

Raw gene expression data was obtained from Elias *et al*. [32]. Our inverse correlation analysis to infer predicted functional miRNA-target relationships is highly dependent on the gene expression patterns, and technical variances were minimized by processing the arrays using the RMA (robust multi-array average) approach [41] implemented in the Bioconductor package affy [42]. RMA normalization consisted of three steps: background correction, probe-level quantile-normalization and probeset summarization using a robust linear model fit to the log-transformed normalized values. Genes exhibiting altered expression were identified using limma package [38] in R.

### Integration of miRNA-mRNA expression data

qPCR-measured miRNAs exhibiting higher absolute fold-changes (absolute log2 fold-change ≥0.7, no p-value threshold) in one or more TamR vs. MCF-7/S0.5 cell lines were selected. To cover a larger and unbiased set of predicted miRNA-target relationships, we considered target predictions made by the following tools: miRanda, miRDB, miRWalk, PICTAR5, RNA22 and Targetscan [43-48]. We next calculated Pearson's correlation coefficient, *r* for expression levels of each predicted miRNA-target pairs. Due to the differences in the sample sizes of relevant cell lines in miRNA and mRNA expression datasets, RMA normalized expression levels of each gene and Cp-values for every miRNA were summarized to one value per cell line type by calculating an average across replicates. Since miRNA expression is expected to be inversely proportional to their mRNA targets and Cp-values from qPCR are inversely proportional to expression levels, a series of tests for correlations between Cp and RMA values were performed over the summarized expression datasets and the resulting correlation coefficients were negated. Highly negatively-correlated (*r* ≤ −0.8) miRNA-mRNA pairs were inferred to have a predicted functional interaction if at least two of the six target predictors supported the interaction.

To assess our hypothesis that the relative extent of changes in expression of the predicted functional targets is higher than that of the non-targets, sets of correlated miRNA-mRNA pairs were tested by two-sample Wilcoxon rank-sum tests for each resistant cell line. Two-sided Wilcoxon tests were performed on the observed fold-changes of predicted functional miRNA targets versus fold-changes of non-targeted mRNAs, including the predicted, but not functional, target mRNAs. P-values of Wilcoxon tests were corrected for multiple testing errors via the Benjamini Hochberg method. If the target mRNAs had lower ranks than the background list of mRNAs, then the log_10_ of Wilcoxon rank sum test p-values were given a negative sign. This analysis was carried out in R statistical environment.

### Identification of tamoxifen resistance-associated miRNAs in tumors of tamoxifen-treated breast cancer patients

The miRNA expression profiles of the cell lines were compared with our previously published dataset (GSE37405) [19] of global miRNA profiles of primary ER+ tumor samples from 152 adjuvant tamoxifen-treated breast cancer patients. This study was comprised of three cohorts, test sets 1, 2 and 3. Test set 1 is a cohort of 52 patients who received an average of 2 years of tamoxifen therapy, had large tumors (12-95 mm) and an average >4.5 axillary lymph nodes with tumor infiltration at the time of diagnosis. Of these patients, 26 later experienced recurrence. Test set 2 consisted of 60 patients with high numbers of tumor-infiltrated lymph nodes (range: 1-29, average: 8.6), 30 of whom experienced recurrence. Test set 3 consisted of 40 patients who had received 3-5 years of tamoxifen therapy, and of these 19 experienced recurrences. Clinical and pathological characteristics of the three patient subgroups are listed in Supplementary Table 8. Normalization and pre-processing of microarray data from GSE37405 [19] was performed in R using *limma* package [38]. The resistant cell lines, TamR1, TamR4 and TamR8, were considered model systems for recurrence, and the parental cell line MCF-7/S0.5 was considered a model for patients without recurrence. Differentially-expressed miRNAs from the cell line model were assessed for possible changes of expression between recurrent and non-recurrent patient samples in the three test sets (cohorts) fitting a logistic regression model. miR-190b, miR-29b, miR-516a-5p, miR-203, expression profiles were used to calculate their predictive power to differentiate recurrent or non-recurrent samples in GSE37405 as a single marker or in combinations. Area under the curve (AUC) values were calculated using the ROCR package [49].

### Gene knockdown studies

Small interfering RNAs (siRNAs) targeting SNAI2, FOXM1 and scrambled siRNA controls were designed and synthesized by Qiagen (Copenhagen, Denmark). Cell transfection was performed using the Ingenio electroporation kit according to manufacturer's instructions (Mirus, Madison, WI, USA). Briefly, cells grown to 70-80% confluence were harvested, counted and resuspended in 100μl of Ingenio electroporation solution containing 150 nM of the required siRNAs. The mixture was transferred into a 0.2 cm cuvette and electroporated using an Amaxa nucleofector device (Lonza, Cologne, Germany). Transfected cells were then seeded in the appropriate culture medium. Cells were harvested 48 or 72 hours later for evaluation of the efficiency of gene knockdown using real time PCR and/or Western blotting [50]. Growth assay was performed 96 hours after gene knockdown. Scrambled siRNA transfected cells were used as controls.

### Evaluation of the effect of specific chemical inhibitors on TamR cell growth

MCF-7/S0.5 and TamR cell lines (10^5^cells/ml) were seeded in 24-well plates (Sigma) in the presence or absence of 1μM tamoxifen (Sigma) and 3μM SNAIL-P53 binding inhibitor GN25 (Millipore, Darmstadt, Germany) or 2.5μM SRC family kinase inhibitor PP2 (Tocris Bioscience, Bristol, UK). Cell growth was measured 3 days after seeding using a crystal violet-based colorimetric assay. Each assay was performed in quadruplicates.

## SUPPLEMENTARY MATERIALS FIGURES AND TABLES







## Abbreviations

DE: differentially expressed
logFC: log2-fold change
