# Supplementary material for: Integrative analysis of miRNA and gene expression reveals regulatory networks in tamoxifen-resistant breast cancer
Source: Oncotarget. 2016 Aug 9;7(35):57239–53. doi: 10.18632/oncotarget.11136 (PMC5302986; doi:10.18632/oncotarget.11136)
Supplement: Supplementary file 1 [file oncotarget-07-57239-s001.pdf]

Integrative analysis of miRNA and gene expression reveals regulatory networks in tamoxifen-resistant breast cancer

SUPPLEMENTARY FIGURES AND TABLES

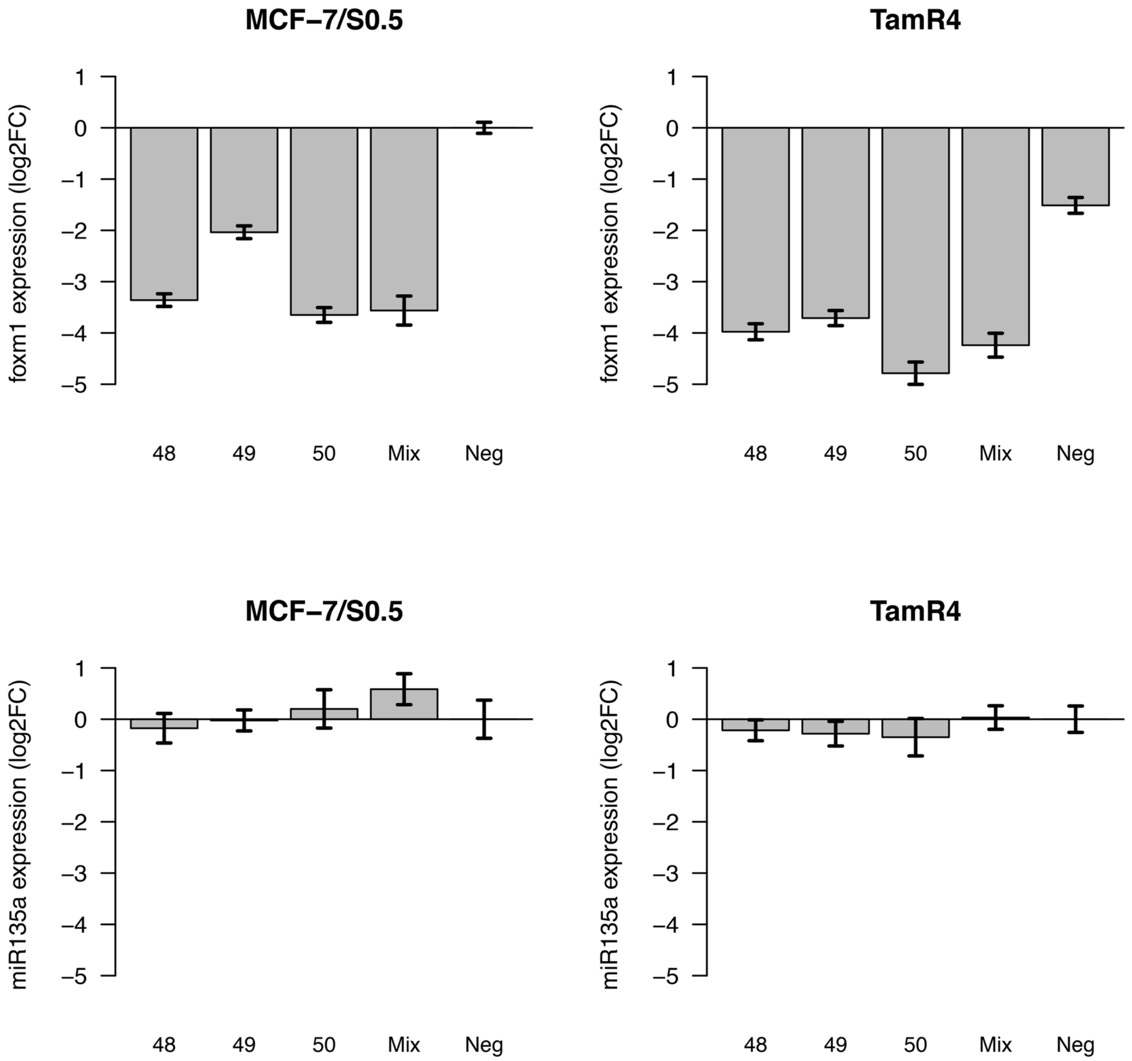

Supplementary Figure 1: Knockdown of FOXM1 identified to exhibit higher expression in MCF-7/S0.5 vs. TamR4 and the effect on miR-135a levels. (top panel) MCF-7/S0.5 and TamR4 cells were transfected with 3 siRNAs specific for FOXM1 either as single siRNAs: 48, 49, 50 or a mixture of all the siRNAs (Mix). All FOXM1-specific siRNAs lead to a statistically significant ($P<0.01$) reduction in gene expression compared with cells transfected with control siRNA (Neg). (lower panel) Knockdown of FOXM1 with specific siRNA vs. negative control lead to no significant alteration of miR-135a expression in both cell lines.

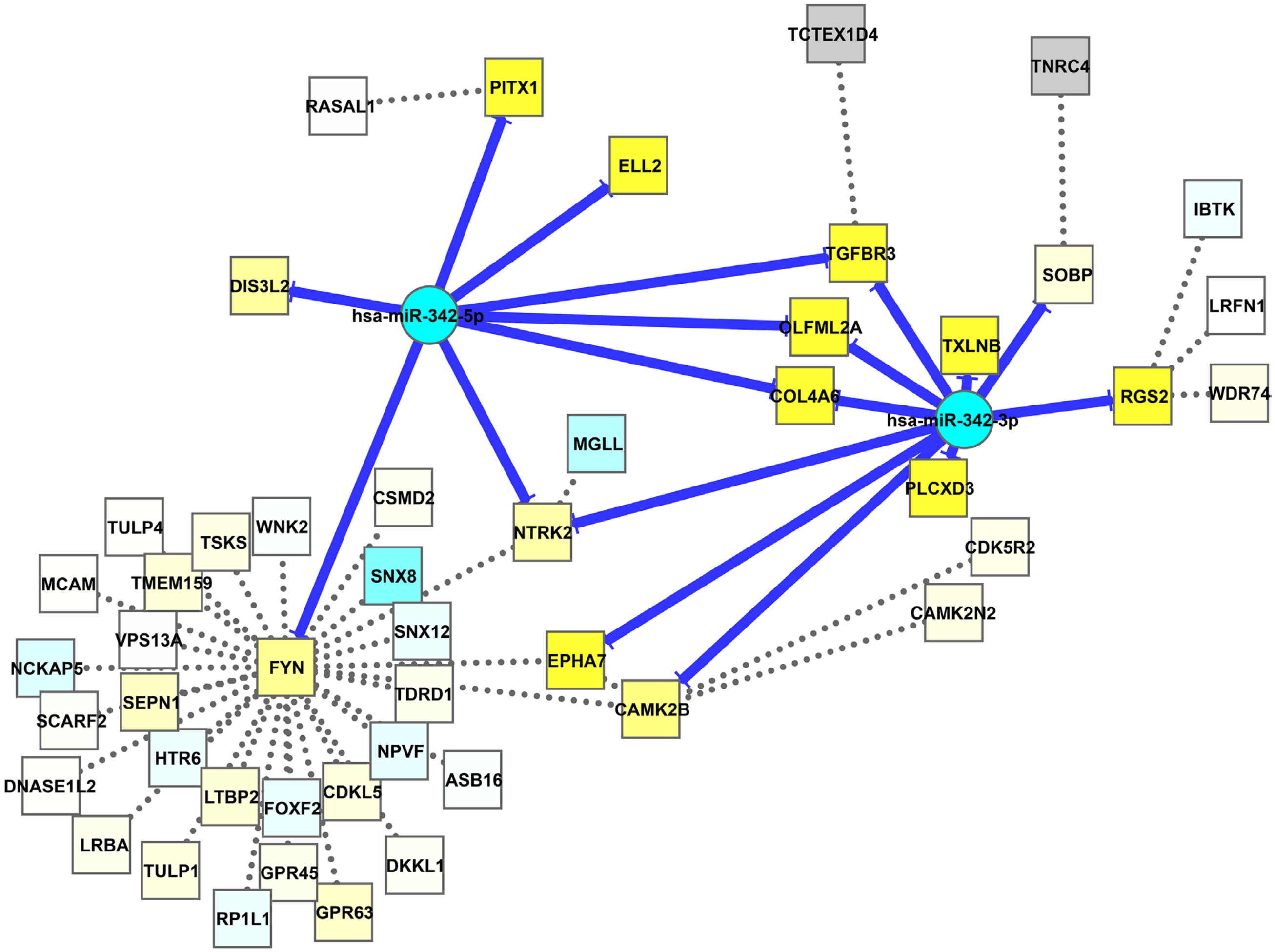

Supplementary Figure 2: miRNA-mediated gene regulation events associated with tamoxifen resistance focusing on miR-342-3p/5p. Fourteen predicted functional targets of miR-342-3p/5p, including *FYN, TGFBR1, COL4A6, CDKN1A*, and Ephrins *EPHA4/7* were identified in the tamoxifen-resistant cell lines. Color and intensity of the nodes correspond to the extent of fold-changes observed in the relevant comparison.

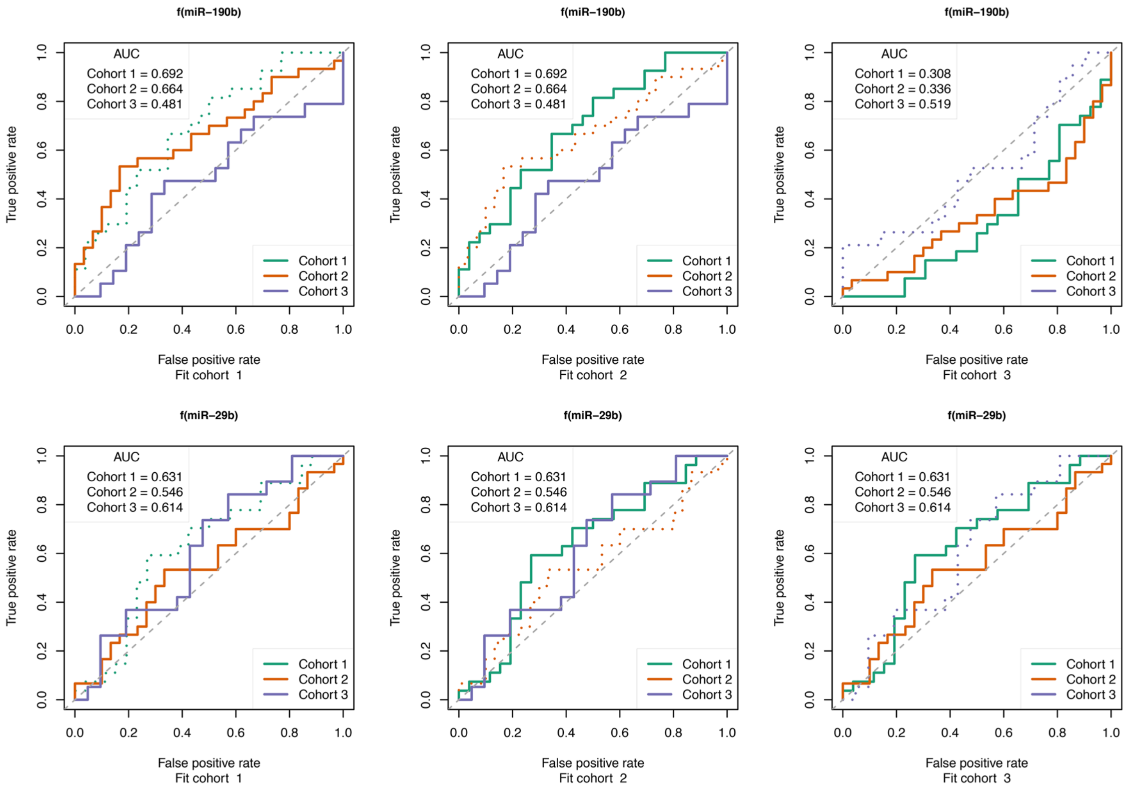

f(miR−190b)
AUC
Cohort 1 = 0.692
Cohort 2 = 0.664
Cohort 3 = 0.481
Cohort 1
Cohort 2
Cohort 3
True positive rate
False positive rate
Fit cohort 1
f(miR−190b)
AUC
Cohort 1 = 0.692
Cohort 2 = 0.664
Cohort 3 = 0.481
Fit cohort 2
f(miR−190b)
AUC
Cohort 1 = 0.308
Cohort 2 = 0.336
Cohort 3 = 0.519
Fit cohort 3
f(miR−29b)
AUC
Cohort 1 = 0.631
Cohort 2 = 0.546
Cohort 3 = 0.614
Fit cohort 1
f(miR−29b)
AUC
Cohort 1 = 0.631
Cohort 2 = 0.546
Cohort 3 = 0.614
Fit cohort 2
f(miR−29b)
AUC
Cohort 1 = 0.631
Cohort 2 = 0.546
Cohort 3 = 0.614
Fit cohort 3
0.0
0.2
0.4
0.6
0.8
1.0

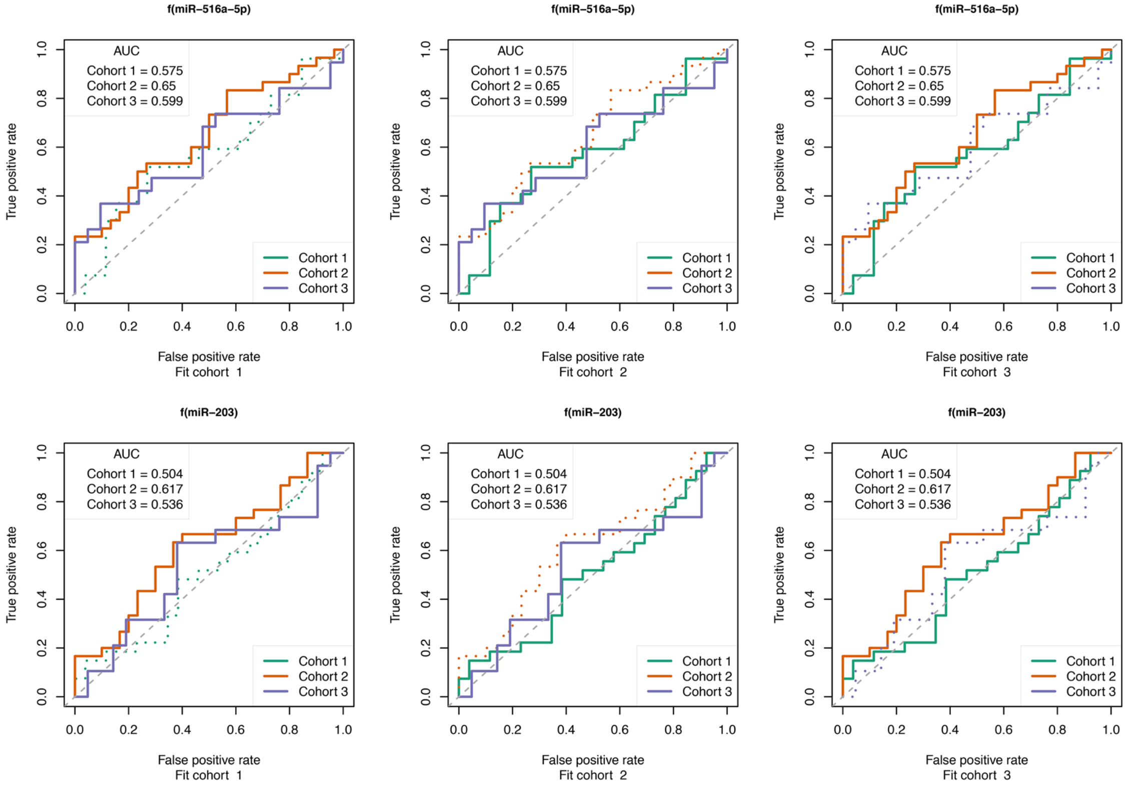

f(miR−516a−5p)
AUC
Cohort 1 = 0.575
Cohort 2 = 0.65
Cohort 3 = 0.599
Cohort 1
Cohort 2
Cohort 3
True positive rate
False positive rate
Fit cohort 1
f(miR−516a−5p)
AUC
Cohort 1 = 0.575
Cohort 2 = 0.65
Cohort 3 = 0.599
Fit cohort 2
f(miR−516a−5p)
AUC
Cohort 1 = 0.575
Cohort 2 = 0.65
Cohort 3 = 0.599
Fit cohort 3
f(miR−203)
AUC
Cohort 1 = 0.504
Cohort 2 = 0.617
Cohort 3 = 0.536
Fit cohort 1
f(miR−203)
AUC
Cohort 1 = 0.504
Cohort 2 = 0.617
Cohort 3 = 0.536
Fit cohort 2
f(miR−203)
AUC
Cohort 1 = 0.504
Cohort 2 = 0.617
Cohort 3 = 0.536
Fit cohort 3
0.0
0.2
0.4
0.6
0.8
1.0

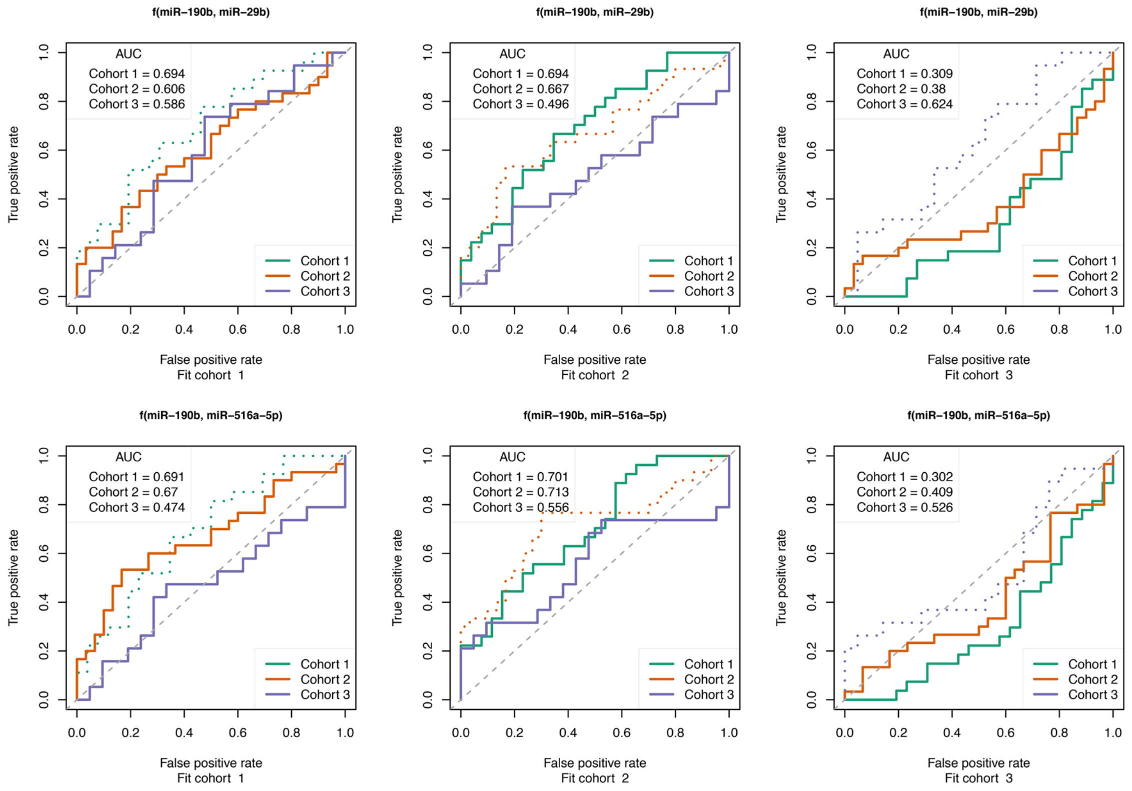
f(miR–190b, miR–29b)
AUC
Cohort 1 = 0.694
Cohort 2 = 0.606
Cohort 3 = 0.586
Cohort 1
Cohort 2
Cohort 3
True positive rate
False positive rate
Fit cohort 1
f(miR–190b, miR–29b)
AUC
Cohort 1 = 0.694
Cohort 2 = 0.667
Cohort 3 = 0.496
Fit cohort 2
f(miR–190b, miR–29b)
AUC
Cohort 1 = 0.309
Cohort 2 = 0.38
Cohort 3 = 0.624
Fit cohort 3
f(miR–190b, miR–516a–5p)
AUC
Cohort 1 = 0.691
Cohort 2 = 0.67
Cohort 3 = 0.474
Fit cohort 1
f(miR–190b, miR–516a–5p)
AUC
Cohort 1 = 0.701
Cohort 2 = 0.713
Cohort 3 = 0.556
Fit cohort 2
f(miR–190b, miR–516a–5p)
AUC
Cohort 1 = 0.302
Cohort 2 = 0.409
Cohort 3 = 0.526
Fit cohort 3

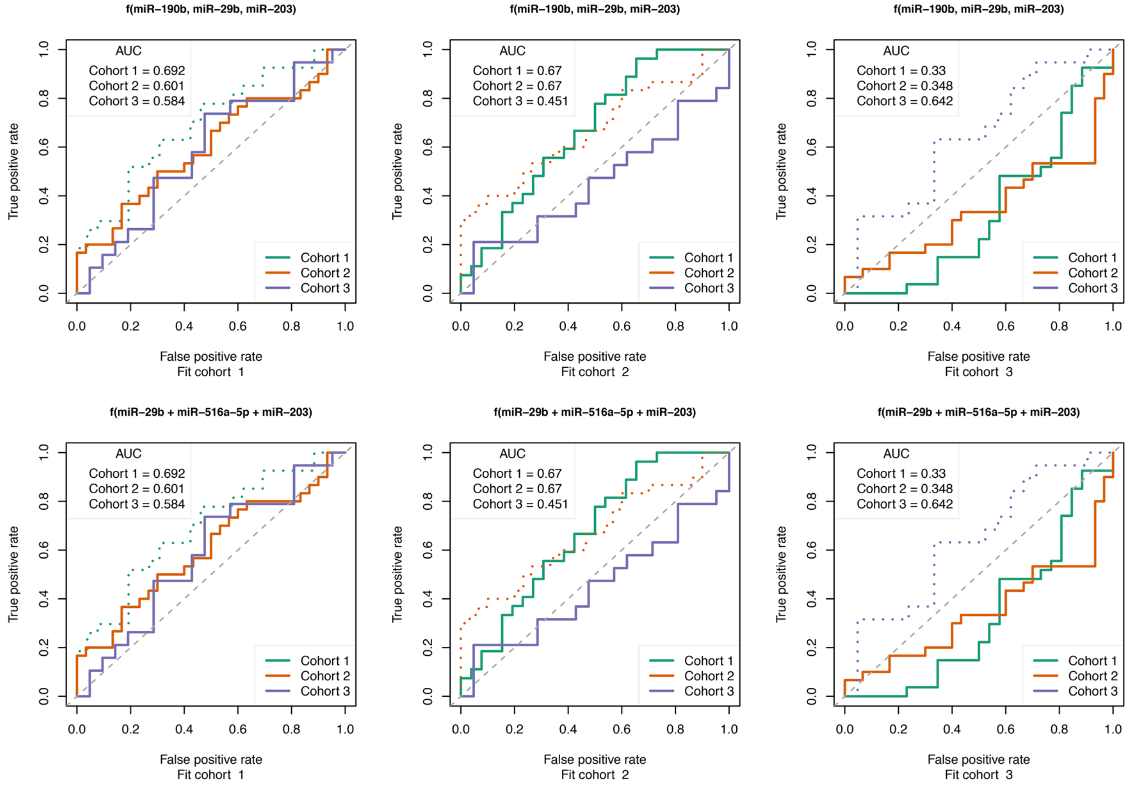
f(miR–190b, miR–29b, miR–203)
AUC
Cohort 1 = 0.692
Cohort 2 = 0.601
Cohort 3 = 0.584
Cohort 1
Cohort 2
Cohort 3
True positive rate
False positive rate
Fit cohort 1
f(miR–190b, miR–29b, miR–203)
AUC
Cohort 1 = 0.67
Cohort 2 = 0.67
Cohort 3 = 0.451
Fit cohort 2
f(miR–190b, miR–29b, miR–203)
AUC
Cohort 1 = 0.33
Cohort 2 = 0.348
Cohort 3 = 0.642
Fit cohort 3
f(miR–29b + miR–516a–5p + miR–203)
AUC
Cohort 1 = 0.692
Cohort 2 = 0.601
Cohort 3 = 0.584
Fit cohort 1
f(miR–29b + miR–516a–5p + miR–203)
AUC
Cohort 1 = 0.67
Cohort 2 = 0.67
Cohort 3 = 0.451
Fit cohort 2
f(miR–29b + miR–516a–5p + miR–203)
AUC
Cohort 1 = 0.33
Cohort 2 = 0.348
Cohort 3 = 0.642
Fit cohort 3

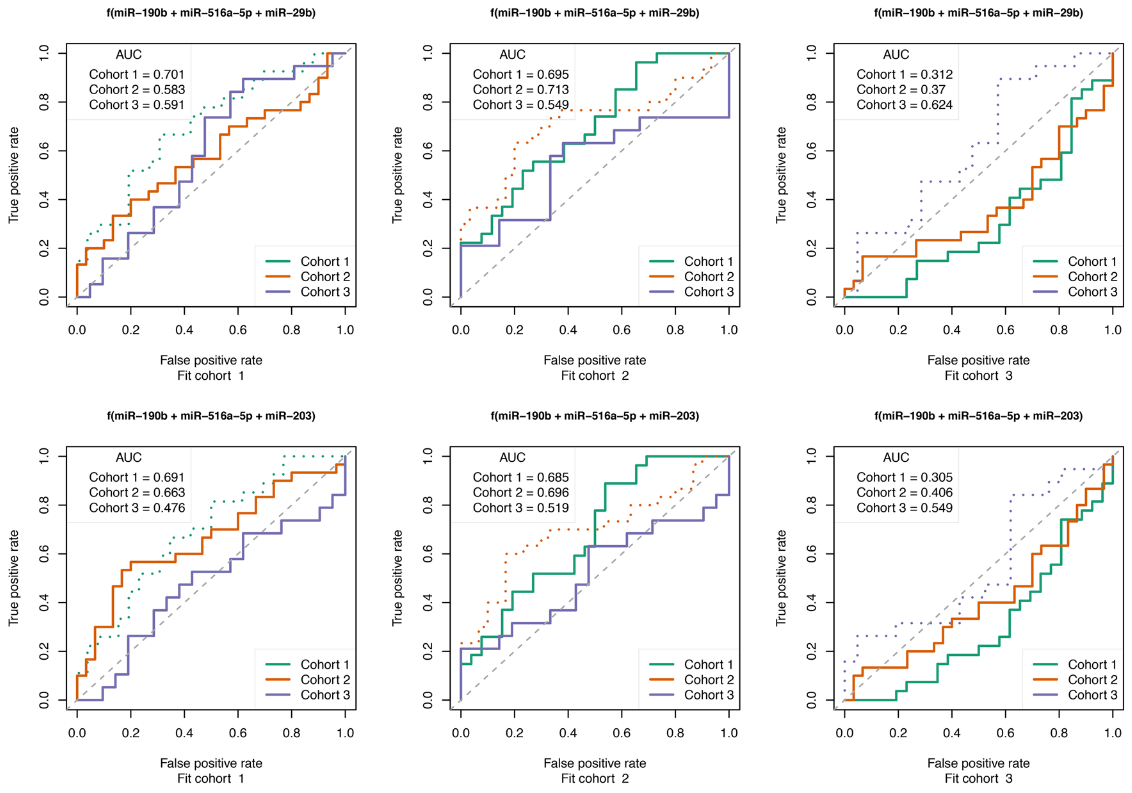

Supplementary Figure 3: ROC curve analysis to assess the prognostic power of miRNAs in an ER+ breast cancer cohort of patients receiving tamoxifen monotherapy. Selected miRNAs that exhibited altered expression in TamRs vs. MCF-7/S0.5 cell lines were also predictive of recurrence following tamoxifen treatment. The differentially expressed miRNA; miR-190b, miR-29b, miR-516a-5p, and miR-203, as single or combinations of miRNAs, were fit on each cohort (Fit cohort) and then applied to all cohorts independently. Training performance is assessed when Fit cohort and test cohorts are the same (dotted lines) while independent test set performance is observed when they differ (solid lines).

Supplementary Table 1: MiRNAs with significantly altered expression profiles in tamoxifen resistant cell lines relative to the parental MCF-7/S0.5 cell line

Contrast (relative to MCF-7/S0.5 cell line)	Differentially expressed miRNAs
TAMR1	miR-101*, miR-1201, miR-1203, miR-1248, miR-130b, miR-130b*, miR-132, miR-135a, miR-135b, miR-142-3p, miR-152, miR-153, miR-181b, miR-182*, miR-18a, miR-1913, miR-192, miR-196a, miR-196b, miR-1979, miR-199b-5p, miR-200a*, miR-203, miR-21, miR-210, miR-212, miR-215, miR-218, miR-22, miR-22*, miR-220b, miR-23b, miR-24-1*, miR-26b*, miR-296-3p, miR-339-5p, miR-33b, miR-345, miR-34b, miR-362-5p, miR-365, miR-378, miR-378*, miR-450a, miR-499-5p, miR-500, miR-501-3p, miR-502-5p, miR-516a-5p, miR-517b, miR-517c, miR-519a, miR-519b-3p, miR-519e, miR-519e*, miR-522, miR-542-5p, miR-551b, miR-577, miR-582-5p, miR-593, miR-598, miR-611, miR-649, miR-652, miR-663b, miR-665, miR-943, miR-95
TAMR4	miR-1, miR-101, miR-101*, miR-106b*, miR-1201, miR-1248, miR-1259, miR-1271, miR-130b, miR-130b*, miR-135a, miR-135b, miR-138, miR-141, miR-141*, miR-143, miR-149, miR-149*, miR-152, miR-153, miR-181a*, miR-181b, miR-181c, miR-183, miR-18a, miR-18a*, miR-18b, miR-190, miR-1908, miR-190b, miR-191*, miR-192*, miR-193a-3p, miR-196a, miR-196b, miR-1979, miR-200a, miR-203, miR-21, miR-210, miR-2113, miR-212, miR-218, miR-22*, miR-26a, miR-26a-2*, miR-26b*, miR-296-3p, miR-29a, miR-29b, miR-29b-1*, miR-29c*, miR-30a, miR-30b, miR-30d, miR-330-3p, miR-337-3p, miR-339-5p, miR-33a*, miR-33b, miR-33b*, miR-342-3p, miR-342-5p, miR-345, miR-365, miR-424, miR-424*, miR-425*, miR-455-5p, miR-499-5p, miR-503, miR-505, miR-516a-5p, miR-517c, miR-519a, miR-519e, miR-520d-3p, miR-522, miR-524-5p, miR-548k, miR-551b, miR-580, miR-582-5p, miR-600, miR-605, miR-622, miR-628-5p, miR-629*, miR-636, miR-649, miR-652, miR-664, miR-665, miR-671-3p, miR-671-5p, miR-7, miR-708, miR-720, miR-760, miR-940, miR-941, miR-95, miR-96
TAMR8	let-7i, let-7i*, miR-101, miR-101*, miR-106b, miR-106b*, miR-1201, miR-1203, miR-1237, miR-1248, miR-126, miR-129*, miR-130b, miR-130b*, miR-135a, miR-135b, miR-142-3p, miR-143, miR-149, miR-152, miR-181b, miR-182, miR-182*, miR-183, miR-18a*, miR-1908, miR-190b, miR-192, miR-194, miR-196a, miR-197, miR-203, miR-205, miR-20b, miR-21*, miR-210, miR-215, miR-218, miR-219-5p, miR-22, miR-22*, miR-23a, miR-23a*, miR-23b, miR-23b*, miR-24-1*, miR-24-2*, miR-26a, miR-27a, miR-29a*, miR-29b-1*, miR-30a, miR-328, miR-335, miR-339-5p, miR-340, miR-342-3p, miR-342-5p, miR-34a*, miR-363, miR-375, miR-378, miR-378*, miR-423-3p, miR-424, miR-449a, miR-450a, miR-455-3p, miR-455-5p, miR-491-5p, miR-503, miR-516a-5p, miR-517c, miR-519a, miR-519e, miR-519e*, miR-551b, miR-574-3p, miR-577, miR-582-5p, miR-590-5p, miR-598, miR-611, miR-625*, miR-628-3p, miR-652, miR-708, miR-720, miR-877*, miR-93, miR-93*, miR-941, miR-942, miR-95, miR-96

Absolute log-ratio of expression ≥0.7 and adjusted p-value ≤0.05.

Supplementary Table 2: Overlap of differentially expressed miRNAs TamR cell lines as measured by qPCR assays

TAMR1 DOWN. TAMR4 DOWN	miR-101*,miR-1201,miR-1248,miR-135a,miR-135b,miR-153,miR-196a,miR-218,miR-33b,miR-499-5p,miR-649,miR-652,miR-95
TAMR1 DOWN. TAMR4 UP	miR-665
TAMR1 DOWN. TAMR8 DOWN	miR-101*,miR-1201,miR-1248,miR-135a,miR-135b,miR-182*,miR-196a,miR-23b,miR-24-1*,miR-378,miR-378*,miR-519e*,miR-577,miR-611,miR-652,miR-95
TAMR1 UP. TAMR4 DOWN	miR-196b,miR-21
TAMR1 UP. TAMR4 UP	miR-130b,miR-130b*,miR-152,miR-181b,miR-18a,miR-1979,miR-203,miR-210,miR-212,miR-22*,miR-26b*,miR-296-3p,miR-339-5p,miR-345,miR-365,miR-516a-5p,miR-517c,miR-519a,miR-519e,miR-522,miR-551b,miR-582-5p
TAMR1 UP. TAMR8 UP	miR-1203,miR-130b,miR-130b*,miR-142-3p,miR-152,miR-181b,miR-192,miR-203,miR-210,miR-215,miR-22,miR-22*,miR-339-5p,miR-450a,miR-516a-5p,miR-517c,miR-519a,miR-519e,miR-551b,miR-582-5p,miR-598
TAMR4 DOWN. TAMR8 DOWN	miR-101,miR-101*,miR-1201,miR-1248,miR-135a,miR-135b,miR-143,miR-183,miR-190b,miR-196a,miR-29b-1*,miR-342-3p,miR-342-5p,miR-424,miR-503,miR-652,miR-708,miR-95,miR-96
TAMR4 DOWN. TAMR8 UP	miR-218,miR-26a,miR-30a
TAMR4 UP. TAMR8 DOWN	miR-106b*
TAMR4 UP. TAMR8 UP	miR-130b,miR-130b*,miR-149,miR-152,miR-181b,miR-18a*,miR-1908,miR-203,miR-210,miR-22*,miR-339-5p,miR-455-5p,miR-516a-5p,miR-517c,miR-519a,miR-519e,miR-551b,miR-582-5p,miR-720,miR-941
TAMR1 DOWN. TAMR4 DOWN. TAMR8 DOWN	miR-101*,miR-1201,miR-1248,miR-135a,miR-135b,miR-196a,miR-652,miR-95
TAMR1 DOWN. TAMR4 DOWN. TAMR8 UP	miR-218
TAMR1 UP. TAMR4 UP. TAMR8 UP	miR-130b,miR-130b*,miR-152,miR-181b,miR-203,miR-210,miR-22*,miR-339-5p,miR-516a-5p,miR-517c,miR-519a,miR-519e,miR-551b,miR-582-5p

Supplementary Table 3: List of miRNAs with altered expression in TamR cell lines as detected by smallRNAseq

See Supplementary File 1

Supplementary Table 4: Overlap and differences between qPCR and sequencing based measures of altered miRNA expression

**Agreement between qPCR and small-RNAseq**	
TAMR1_up	miR-130b, miR-130b*, miR-132, miR-152, miR-196b, miR-1979, miR-203, miR-212, miR-22, miR-26b*, miR-339-5p, miR-345, miR-450a, miR-501-3p, miR-516a-5p, miR-522, miR-542-5p, miR-598
TAMR1_down	miR-1201, miR-135a, miR-196a, miR-218, miR-23b, miR-24-1*, miR-378, miR-499-5p, miR-652, miR-95
TAMR4_up	miR-1271, miR-130b*, miR-149, miR-152, miR-18a, miR-18a*, miR-18b, miR-191*, miR-203, miR-210, miR-22*, miR-26b*, miR-339-5p, miR-33a*, miR-33b*, miR-345, miR-365, miR-516a-5p, miR-519a, miR-522, miR-551b, miR-580, miR-629*, miR-636, miR-671-3p, miR-671-5p, miR-760, miR-940, miR-941
TAMR4_down	miR-101, miR-1201, miR-1259, miR-135a, miR-141, miR-143, miR-181c, miR-183, miR-190, miR-190b, miR-196a, miR-218, miR-26a, miR-29a, miR-29b-1*, miR-30a, miR-30b, miR-30d, miR-330-3p, miR-342-3p, miR-342-5p, miR-424, miR-424*, miR-503, miR-548k, miR-652, miR-708, miR-95, miR-96
TAMR8_up	let-7i*, miR-130b*, miR-142-3p, miR-149, miR-152, miR-1908, miR-197, miR-203, miR-20b, miR-210, miR-22, miR-22*, miR-339-5p, miR-363, miR-375, miR-449a, miR-455-3p, miR-519a, miR-582-5p, miR-598, miR-628-3p, miR-941
TAMR8_down	miR-1201, miR-143, miR-190b, miR-196a, miR-29b-1*, miR-335, miR-342-5p, miR-378, miR-424, miR-652, miR-942, miR-95
**Differences between qPCR and small-RNAseq**	
Altered expression by small-RNAseq but not by qPCR	miR-1252, miR-127-3p, miR-206, miR-217, miR-224, miR-338-5p, miR-379, miR-491-3p, miR-492, miR-508-3p, miR-548j, miR-548l, miR-549, miR-556-3p, miR-573, miR-891a, miR-124*, miR-145*, miR-146a, miR-155*, miR-302a*, miR-302b, miR-302c, miR-372, miR-411, miR-518b, miR-548h, miR-370, miR-432, miR-449b, miR-514, **miR-105, miR-10a, miR-10b, miR-122, miR-184, miR-34c-5p, miR-452, miR-556-5p, miR-886-5p, miR-361-5p, miR-486-3p, miR-103-as, miR-144***

(absolute logFC ≥0.7, p ≤0.05, where applicable). MiRNAs listed in bold failed the QC by qPCR (Cp >37).

Supplementary Table 5: Highly relevant functional miRNA-target relationship inferred by inverse-correlation analysis. The miRNA-target pairs in the table were selected from the complete list of functional miRNA-targets due to their significant expression changes in the tamoxifen-resistant cell lines vs. MCF-7/S0.5 cell line

See Supplementary File 2

Supplementary Table 6: Functional targets of miR-135b identified by inverse correlation analysis

miRNA	Functional targets
miR-135b	TOM1L1, ATP9A, HIPK3, ACTR1A, NAMPT, G3BP1, MBNL2, TRIM13, BCAS2, BTN2A2, AGR2, ARFGEF2, SORBS1, EXOC5, GNA13, GNB5, ADCY1, NFAT5, TOB2, ZBTB6, FRS2, TMED10, ASCC3, MAPRE2, DIDO1, POLR3A, HHLA3, AKAP13, NXPH3, PRR4, CYP2U1, PIK3IP1, SLC2A13, KCTD12, ZFYVE27, ANKRD22, SLC15A4, CLTC, ZNF440, CMPK2, ACVR1C, COPA, CPD, ASB5, C20orf112, SMCR8, C10orf46, CSNK1A1, ZFP90, TOM1L2, WIPF2, SLC30A7, CNIH3, C2orf67, PRELID2, DAG1, CITED4, DDX3X, PRICKLE2, DCP2, DHX36, SYNPO2, DMRT1, ABAT, DUSP3, MEGF9, EIF5, CADM4, ELK3, TMEM192, EPHA4, HIPK1, CLN8, ABCD1, FANCF, ATAD3C, GPR115, C6orf89, FKBP1A, IKZF2, PHLDA1, ZNF652, ENPP4, CLSTN1, FOXJ3, ZNF365, DIS3, MAPRE1, DIP2C, PCNX, MAST3, XPO7, KIF21B, NMNAT2, SETX, TNRC6B, HIC2, GPATCH8, SMC5, DCUN1D4, JMJD6, PACS2, KIAA0556, IQCE, FBXW11, KIAA1033, DPY19L1, CRTC1, EXOSC2, RYBP, RHOQ, ABCA5, BACE1, YTHDF3, RNF144B, GALC, ARIH1, ZNF385A, KBTBD2, C1orf144, CACNG4, AFF4, VPS4A, ZNF330, GLS, GM2A, BCL9L, PGM2L1, NUDT7, PTRF, GDPD1, ZIK1, CYP4V2, OSTM1, C16orf72, ORMDL2, GTF2F1, TMOD2, HADHA, HAS3, HIF1A, APC, ACADL, RGAG4, ZC3H12B, IL16, TRIM23, FAM111B, KCNG1, KCNS1, KCNS3, YPEL2, LASP1, LDLR, LIPA, LMAN1, LTBP2, ARNTL, CAPRIN1, SMAD3, SMAD5, ARSD, MB, MBNL1, MCL1, CD46, MAP3K3, RAB8A, MLL, MOV10, MTF1, MTHFR, NAB1, NAP1L2, ACLY, NEO1, NF1, NFX1, NOTCH2, NPAS2, OAS3, OGDH, ORC5L, ARHGEF3, F11R, PCDHGC3, TMED5, PCDH9, PHF20, MRPL30, ZNF117, CDC40, NIP7, POLK, ZNF107, PGGT1B, PIK3C2A, PI4KB, PIP4K2A, PITX2, ATP6V0A1, CSNK1G1, PION, KRT20, TMCO1, ATRX, MED1, PPIA, DNAJB12, MBTD1, PGPEP1, TMEM104, RNF43, WHSC1L1, ZNF434, C14orf119, C17orf80, PLEKHB2, VPS37C, PDPR, ELP3, PNPO, PPP2R1B, SETD5, SMU1, PCMTD2, ASXL2, SYNJ2BP, TBC1D2, PI4K2A, PPT1, GALNT10, BTBD2, TMEM127, PRPF40A, PRKCA, TRERF1, TEX2, CTTNBP2NL, MAPK1, PCDHGB5, PCDHGA10, PCDHGA1, PCDHB13, PCDHB10, MOSPD1, ZNF253, EIF5A2, BARHL1, CCDC47, TMEM159, ATP10D, LYRM2, KIAA1161, INTS2, NUFIP2, ZNF398, KLHL8, TMEM181, HOMEZ, FNIP2, POGK, EPB41L5, ZNF317, PCDHB16, RBAK, PTPN9, PTPRE, PURB, BCAT1, RAB6A, RALB, RAP2A, PLEKHA2, RELA, RIT1, ABCE1, ZFAND3, ROCK1, RPS6KB1, RRBP1, RSU1, TSPAN31, SC5DL, SDC1, SDC2, SDCBP, ZNF335, SEC14L1, SEL1L, ZFYVE20, TMEM168, SGCB, SH3BGRL, CLEC7A, SLC5A3, SLC7A2, PCP4L1, SLC9A2, SNTB2, SOLH, SOS2, SPINK2, STAT1, SUOX, SYK, HNF1B, TEAD1, TEP1, TFAP2A, TIMP2, TRPC1, CAPN5, UBA52, UBE2D3, UBTF, UVRAG, VIPR1, BEST1, ZKSCAN1, ZSCAN21, ZNF91, ZNF136, ZNF223, ZNF229, OTUB2, PCYOX1L, C19orf42, FYCO1, SH3TC2, SCRN3, DHX40, HHIPL2, QSER1, RPAP2, NRSN2, OPA3, ZNF430, EDEM3, CYB5B, RND2, SNX27, USP9X, TMEM47, TM2D2, PIP5K1A, DTNBP1, RNASEH2C, FYTTD1, C14orf142, ADO, DIRC2, PIK3R3, API5, DISP2, CASK, RUNX2, NCOA1, STX16, SYNJ1, SQSTM1, SLC5A6, PRPF4B, STBD1, CDKL2, EMILIN3, ATP6V0D1, SLC39A13, CHURC1, ZMYM6, PTER, TRIP11, UBE4A, ARHGAP18, KIF3B, ZRANB2, ORMDL3, TP53INP1, MED23, ROCK2, CHST10, SLC4A7, ENTPD1, SEC22B, H6PD, ENTPD4, CD58, CD59, WSCD2, CLINT1, KIAA0562, KIAA0513, KIAA0494, SPATA2, ZBTB24, PLEKHM1, CEP350, TOX4, HELZ, MED13, SLC12A6

Supplementary Table 7: Potential influence of the differentially expressed miRNA on mRNA expression levels

miRNA, mRNA change	Cell line	DE miRNAs	DE miRNA w/Corr. Targets *	DE mRNA	DE mRNA under miRNA-regulation	% mRNAs under miRNA-regulation**	Odds ratio
Up, Down	TamR1	**40**	31	**67**	44	65.7	0.71
	TamR4	**51**	33	**231**	140	60.6	0.65
	TamR8	**47**	39	**22**	12	54.5	0.48
Down, Up	TamR1	**29**	26	**204**	132	64.7	0.88
	TamR4	**52**	41	**205**	145	70.7	0.63
	TamR8	**48**	33	**125**	79	63.2	0.6

Footnote: Direction of change of expression of miRNA/mRNA expression in a TamR cell line is relative to MCF-7/S0.5.
* refers to the functional miRNA-target relationship;
** refers to functional targets exhibiting differential expression in the same TamR.
DE; differentially expressed. Corr targets; correlated miRNA targets.

Supplementary Table 8: Characteristics of included patients and their breast cancer tumor (N=152)

	**Discovery set**		**Test set #1**		**Test set #2**	
	**R (n=26)**	**N (n=26)**	**R (n=30)**	**N (n=30)**	**R (n=19)**	**N (n=21)**
**Age** Avg. (range), years	59.2 (48-73)	62.8 (49-74)	61.5 (49-70)	62.7 (52-72)	59.1 (50-72)	60 (49-72)
**Size** Avg. (range), mm	33.19 (12-85)	30.1 (14-95)	31.4 (7-80)	26.2 (8-58)	28.4 (8-65)	20.5 (10-45)
**Positive lymph nodes** Avg (range)	4.7 (0-13)	4.5 (0-13)	8.6 (1-29)	3.0 (0-14)	3.3 (0-11)	1.6 (0-8)
**ER status** (avg.%/median %)*	91.4/95	85.9/100	77.9/90	83.9/83	82.4/92.5	83.1/90
Positive**	4	1	7	12	6	5
Negative†	1	0	1	3	0	0
Unknown	0	2	1	3	1	0
**PgR status** (avg.%/median %)*	73.2/80	81.3/90	65/72.5	70.2/80	32.9/15	36.8/10
Positive**	3	1	9	9	1	-
Negative	6	7	11	5	2	6
Unknown	0	2	2	1	5	6
**Diagnosis** (IDC/ILC/unknown)	20/6/0	20/6/0	25/4/1	26/3/1	15/1/3	18/0/3
**Tamoxifen** Avg (range), years	1.8 (0.3-5.0)	2.1 (0.7-5.0)	1.7 (0.8-5.2)	1.6 (0.8-5.3)	3.1 (1.7-5.0)	4.5 (2.7-5.0)
**TTR** Avg (range), years	3.4 (0.7-8.7)	–	3.7 (0.8-14.9)	–	3.6 (0.8-8.7)	–

*: the average and median were calculated only for the tumors defined as positive, i.e. staining was observed in ≥ 10 % of tumor cells by immunohistochemistry.
**: If the actual percentage was not provided, patients were deemed positive if ER staining was observed in ≥ 10 % of tumor cells by immunohistochemistry and/or target protein (ER or PgR) was > 10 fmol/mg total protein as determined by biochemistry.
†: the 5 ER- tumors had a PgR status of 90 %, 50 %, 90 %, 80 % and IHC+ (i.e. > 10 %) respectively.
R: patients with recurrence. N: patients without recurrence. TTR: time to recurrence.
